# Prosody in the hands of the speaker

**DOI:** 10.3389/fpsyg.2014.00700

**Published:** 2014-07-07

**Authors:** Bahia Guellaï, Alan Langus, Marina Nespor

**Affiliations:** ^1^Laboratoire Ethologie, Cognition, Développement, Département de Psychologie, Université Paris Ouest Nanterre La DéfenseNanterre, France; ^2^Language Cognition and Development Laboratory, Cognitive Neuroscience Sector, International School for Advanced StudiesTrieste, Italy

**Keywords:** gestures, comprehension, speech perception, ambiguity, prosody

## Abstract

In everyday life, speech is accompanied by gestures. In the present study, two experiments tested the possibility that spontaneous gestures accompanying speech carry prosodic information. Experiment 1 showed that gestures provide prosodic information, as adults are able to perceive the congruency between low-pass filtered—thus unintelligible—speech and the gestures of the speaker. Experiment 2 shows that in the case of ambiguous sentences (i.e., sentences with two alternative meanings depending on their prosody) mismatched prosody and gestures lead participants to choose more often the meaning signaled by gestures. Our results demonstrate that the prosody that characterizes speech is not a modality specific phenomenon: it is also perceived in the spontaneous gestures that accompany speech. We draw the conclusion that spontaneous gestures and speech form a single communication system where the suprasegmental aspects of spoken language are mapped to the motor-programs responsible for the production of both speech sounds and hand gestures.

## Introduction

Human language is a multimodal experience: it is perceived through both ears and eyes. When perceiving speech, adults automatically integrate auditory and visual information (McGurk and MacDonald, 1976), and seeing someone speaking may improve speech intelligibility (Sumby and Pollack, 1954). The visual information involved in speech is not limited to the lips, the mouth and the head, but can also involve other cues such as eyebrow movements (Bernstein et al., 1998; Graf et al., 2002; Krahmer and Swerts, 2004; Munhall et al., 2004). In fact, in face-to-face interactions people use more than their voice to communicate: the whole body is involved and may serve informative purposes (Kendon, 1994; Kelly and Barr, 1999 for a review). For example, when interacting with others, people all around the world usually also produce spontaneous gestures while talking. In fact gestures are so connected with speech that people may be found gesturing when nobody sees them (Corballis, 2002) and even congenitally blind people gesture when interacting with each other (Iverson and Goldin-Meadow, 1998). Yet, the role of gestures that accompany speech (i.e., co-speech gestures) in communication is still not well understood and little if any attention to the relation between co-speech gestures and the syntactic and prosodic structure of spoken language has been paid in previous studies. Some authors claim that these co-speech gestures are not produced to serve any communicative purposes (Rimé and Shiaratura, 1991). On the contrary, others suggest that gestures and speech are parts of the same system and are performed for the purpose of expression (Kendon, 1983; McNeill, 1992). One way to understand the implication of co-speech gestures in communication is to study their implications at the different levels of the utterance. The present study aimed to investigate the role of gestures that accompany speech at the prosodic level in speech perception.

Gestures accompanying speech are known to ease the speaker's cognitive load, and gesturing helps solving diverse individual tasks ranging from mathematics to spatial reasoning (Cook and Goldin-Meadow, 2006; Chu and Kita, 2011). Gestures are also believed to promote learning in adults as well as in children (Ping and Goldin-Meadow, 2010), to aid the conceptual planning of messages (Alibali et al., 2000), and to facilitate lexical access (Alibali et al., 2000). This suggests that gestures that accompany speech might maximize information about events by providing it cross-modally (de Ruiter et al., 2012). In fact, human infants' canonical babbling is temporally related to rhythmic hand activity already at 30 weeks of age (Locke et al., 1995), suggesting that gestures and speech go “hand-in-hand” from the earliest stages of cognitive development (McNeill, 1992; So et al., 2009).

Here we investigate whether gestures also convey some information about the prosodic structure of spoken language. We test whether prosody, an essential aspect of language, is also detected in gestures. In the auditory modality, prosody is characterized by changes in duration, intensity and pitch (for an overview see Cutler et al., 1997; Warren, 1999; Speer and Blodgett, 2006; Langus et al., 2012). Speakers can intentionally manipulate these acoustic cues to convey information about their states of mind (e.g., irony or sarcasm), to define the type of speech act they are making (e.g., a question or an assertion), and to highlight certain elements over others (e.g., by contrasting them). Importantly, prosody also conveys information about the structure of language. Because the grammatical structure of human language is automatically mapped onto prosodic structure during speech production (Langus et al., 2012), the prosody of spoken language also signals the grammatical structure (Nespor and Vogel^1^, 1986, 2007). Though prosody offers cues to different aspects of grammar, here we concentrate on the role of prosody in conveying information about syntactic structure.

It has been observed that prosodic cues are the most reliable cues for segmenting continuous speech cross-linguistically (Cutler et al., 1997). Adult listeners can use these cues to constrain lexical access (Christophe et al., 2004), to locate major syntactic boundaries in speech (Speer et al., 2011), and to determine how these relate to each other in sentences (Fernald and McRoberts, 1995; Langus et al., 2012). This is best seen in cases where listeners can disambiguate sentences that have more than one meaning (e.g., [bad] [boys and girls] vs. [bad boys] [and girls]) by relying on prosody alone (Lehiste et al., 1976; Nespor and Vogel, 1986, 2007; Price et al., 1991). Manipulations of the prosodic structure influence how listeners interpret syntactically ambiguous utterances (Lehiste, 1973; Lehiste et al., 1976; Cooper and Paccia-Cooper, 1980; Beach, 1991; Price et al., 1991; Carlson et al., 2001; see Cutler et al., 1997). These effects of prosody emerge quickly during online sentence comprehension, suggesting that they involve a robust property of the human parser (Marslen-Wilson et al., 1992; Warren et al., 1995; Nagel et al., 1996; Pynte and Prieur, 1996; Kjelgaard and Speer, 1999; Snedeker and Trueswell, 2003; Weber et al., 2006). Naive speakers systematically vary their prosody depending on the syntactic structure of sentences and naive listeners can use this variation to disambiguate utterances that—though containing the same sequence of words—differ in that they are mapped from sentences with different syntactic structures (Nespor and Vogel, 1986, 2007; Snedeker and Trueswell, 2003; Kraljic and Brennan, 2005; Schafer et al., 2005). These studies indicate that users of spoken language share implicit knowledge about the relationship between prosody and syntax and that they can use both during speech production and comprehension. To account for the syntax-prosody mapping, Nespor and Vogel (1986, 2007) have proposed a hierarchy that at the phrasal level contains—among other constituents—the Phonological Phrase (PP) and the Intonational Phrase (IP). These constituents are signaled in different ways: besides being signaled through external sandhi rules that are bound to a specific constituent, the PP right edge is signaled through final lengthening, and the IP level is signaled through pitch resetting at the left edge and through final lengthening at the right edge.

Here we ask whether prosody could also be perceived visually in the spontaneous gestures that accompany speech. In English and Italian, specific hand gestures ending with an abrupt stop, called “beats” (i.e., McNeill, 1992), are temporally related to pitch accents in speech production (Yasinnik et al., 2004; Esposito et al., 2007; Krahmer and Swerts, 2007). Also in sign languages, prosodic cues are not only conveyed through facial expressions, but also through hand and body movements (Nespor and Sandler, 1999; Wilbur, 1999; Sandler, 2011; Dachkovsky et al., 2013). A model developed on the basis of Israeli Signed Language showed that body positions align with rhythmic manual features of the signing stream to mark prosodic constituents' boundaries at different levels of the prosodic hierarchy (Nespor and Sandler, 1999; Sandler, 1999, 2005, 2011). More recently, Sandler (2012) proposed that many actions of the body in sign languages—that she calls “dedicated gestures”—perform linguistic functions and contribute to prosodic structure.

Do people perceive prosody and co-speech gestures as a coherent unit in everyday interactions? There is some evidence that both adults and infants match the global head and facial movements of the speaker with speech sounds (Graf et al., 2002; Munhall et al., 2004; Blossom and Morgan, 2006; Guellaï et al., 2011). However, it is unknown whether visual prosodic cues that accompany speech, but are not directly triggered by the movements of the vocal tract, are actually used to process the structure of the speech signal. Here we ask whether prosody can be perceived in the spontaneous gestures of a speaker (Experiment 1), and if listeners can use gestures to disambiguate sentences with the same sequence of words mapped onto different speech utterances that have two alternative meanings (Experiment 2). To investigate which prosodic cues participants rely on in disambiguating these sentences, we constructed sentences where disambiguation could be either due to IP or to PP boundaries. This enabled us to test whether the prosodic hierarchy is discernable from gestures alone.

## Experiment 1

In this first experiment, we explored whether gestures carry prosodic information. We tested Italian-speaking participants in their ability to discriminate audio-visual presentations of low-pass filtered Italian utterances where the gestures either matched or mismatched the auditory stimuli (Singer and Goldin-Meadow, 2005). While low-pass filtering renders speech unintelligible, it preserves the prosody of the acoustic signal (Knoll et al., 2009). This guaranteed that only prosodic information was available to the listeners.

### Methods

#### Participants

We recruited 20 native speakers of Italian (15 females and 5 males, mean age 24 ± 5) from the subject pool of SISSA—International School of Advanced Studies (Trieste, Italy). Participants reported no auditory, vision, or language related problems. They received monetary compensation.

#### Stimuli

We used sentences that contain the same sequence of words and that can be disambiguated using prosodic cues at one of two different levels of the prosodic hierarchy. The disambiguation could take place at the IP level—the higher of these two constituents, coextensive with intonational contours—signaled through pitch resetting and final lengthening (Nespor and Vogel, 1986, 2007). For example, in Italian, *Quando Giacomo chiama suo fratello è sempre felice* is ambiguous because depending on the IP boundary *è sempre felice ((he) is always happy*) could refer to either *Giacomo* or *suo fratello* (*his brother*): (1) [Quando Giacomo chiama]_IP_ [suo fratello è sempre felice]_IP_ (*When Giacomo calls him his brother is always happy*); or (2) [Quando Giacomo chiama suo fratello]_IP_ [è sempre felice]_IP_ (*When Giacomo calls his brother he is always happy*).

Alternatively, the disambiguation could take place at the PP level where phrase boundaries are signaled through final lengthening. The PP extends from the left edge of a phrase to the right edge of its head in head-complement languages (e.g., Italian and English); and from the left edge of a head to the right edge of its phrase in complement-head languages (e.g., Japanese and Turkish) (Nespor and Vogel, 1986, 2007). An example of a phrase with two possible meanings is *mappe di città vecchie* that is ambiguous in Italian because depending on the location of the PP boundaries, the adjective *vecchie* (*old*) could refer to either *città* (towns) or *mappe* (maps): (1) [mappe di città]_PP_ [vecchie]_PP_ (old *maps of towns*); or (2) [mappe]_PP_ [di città vecchie]_PP_ (*maps of old towns*) (for more details see the list of the sentences ambiguous at the IP and PP levels used in Experiments 1 and 2 in Table 1). The presentation of the two types of sentences—those ambiguous at the IP level and those ambiguous at the PP level—was randomized across subjects.

**Table 1:** **Sentences ambiguous at the IP or PP level used in both Experiments with their prosodic parsing and their two possible meanings translated in English**.

**Sentences ambiguous at the Intonational Phrase level (IP)**	**Sentences ambiguous at the Phonological Phrase level (PP)**
[[Alla conferenza]_PP_ [Luciano]_PP_ [ha parlato naturalmente]_PP_]_IP_	[[Come hai visto]_PP_]_IP_ [[la vecchia]_PP_ [legge]_PP_ [la regola]_PP_]_IP_
At the conference Luciano has talked in a natural way	As you see the old woman reads the rule
[[Alla conferenza]_PP_ [Luciano]_PP_ [ha parlato]_PP_]_IP_ [[naturalmente]_PP_]_IP_	[[Come hai visto]_PP_]_IP_ [[la vecchia legge]_PP_ [la regola]_PP_]_IP_
Of course Luciano talked at the conference	As you see the old law rules it
[[Come ti avevo detto]_PP_]_IP_ [quando Giorgio]_PP_ [chiama]_*PP*_]_IP_ [[suo fratello]_PP_ [è sempre nervoso]_PP_]_IP_	[[Come sicuramente hai visto]_PP_]_IP_ [la vecchia]_PP_ [sbarra]_PP_ [la porta]_PP_]_IP_
	As you for sure have seen the old lady blocks the door
As I had told you when Giorgio calls his brother he is always happy	[[Come sicuramente hai visto]_PP_]_IP_ [[la vecchia]_PP_ [sbarra]_PP_ [la porta]_PP_]_IP_
[[Come ti avevo detto]_PP_]_IP_ [quando Giorgio]_PP_ [chiama]_PP_][suo fratello]_PP_ [è sempre nervoso]_PP_]_IP_	As you for sure have seen the old bar carries it
As I had told you when Giorgio calls his brother is always happy	
[[Come hai visto]_PP_]_IP_ [[quando Luca]_PP_ [chiama]_PP_ [il suo gatto]_PP_]_IP_ [è sempre felice]_PP_]	[[Come ti avevo detto]_PP_]_IP_ [[quando Luca]_PP_ [legge Dante]_PP_ [è felice]_PP_]_IP_
As you have seen when Luca calls his cat he is always happy	As I had told you when Luca reads Dante he is happy
[[Come hai visto]_PP_]_IP_ [[quando Luca]_PP_ [chiama]_PP_]_IP_ [[il suo gatto]_PP_ [è sempre felice]_PP_]_IP_	[[Come ti avevo detto]_PP_]_IP_ [[quando Luca]_PP_ [legge]_PP_ [Dante]_PP_ [è felice]_PP_]_IP_
As you have seen when Luca calls his cat is always happy	As I had told you when Luca reads Dante is happy
[[Come ti avevo detto]_PP_]_IP_ [[se Giacomo]_PP_ [scrive bene]_PP_ [è felice]_PP_]_IP_	[[Sanno]_PP_ [tutti]_PP_ [che canta solo]_PP_ [se è felice]_PP_]_IP_
As I had told you if Giacomo writes well he is happy	Everybody knows that he sings alone if he is happy
[[Come ti avevo detto]_PP_]_IP_] [[se Giacomo]_PP_ [scrive]_PP_]_IP_] [[Bene]_PP_ [è felice]_PP_]_IP_	[[Sanno]_PP_ [tutti]_PP_ [che canta]_PP_ [solo]_PP_ [se è felice]_PP_]_IP_
	Everybody knows that he sings only if he is happy
As I had told you if Giacomo calls Bene is happy	
[[Sai]_PP_ [che parla]_PP_ [molte lingue]_PP_ [naturalmente]_PP_]_IP_	
You know that he speaks many languages in a natural way	
[[Sai]_PP_ [che parla]_PP_ [molte lingue]_PP_]_IP_ [[naturalmente]_PP_]_IP_]	
You of course know that he speaks many languages	
[Come ti avevo detto]_PP_]_IP_ [salta]_PP_ [il muro]_PP_ [più alto]_PP_ [naturalmente]_PP_]_IP_	
As I had told you s/he jumps over the tallest wall in a natural way	
[Come ti avevo detto]_PP_]_IP_ [salta]_PP_ [il muro]_PP_ [più alto]_PP_]_IP_ [[naturalmente]_PP_]_IP_	
As I had told you of course s/he jumps over the tallest wall	

**Figure 1:**
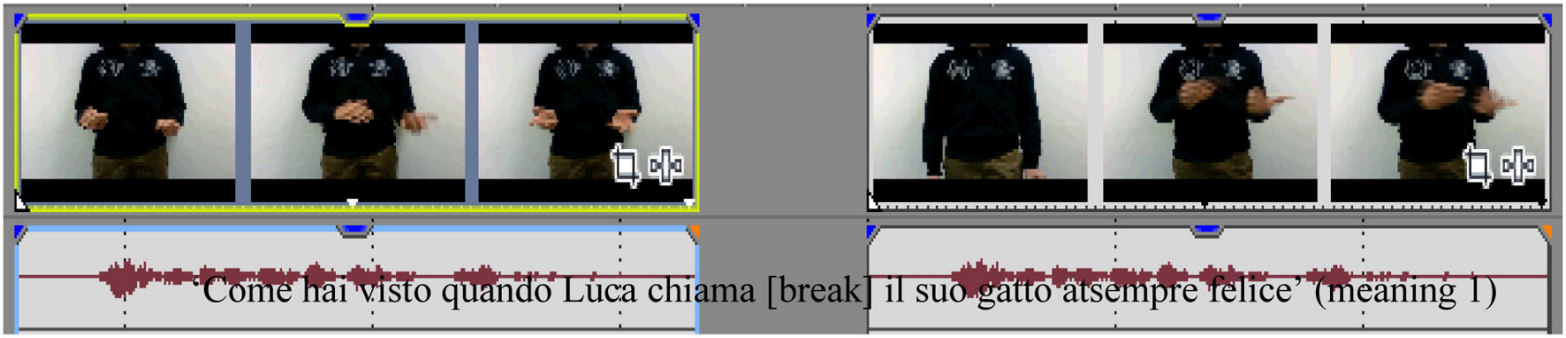
**Examples of the stimuli used in both Experiments (i.e., with speech being filtered for Experiment 1)**. Here the sentence is “Come hai visto quando Luca chiama il suo gatto è sempre felice.” Two meanings are possible: “As you have seen when Luca calls his cat is always happy” (meaning 1) vs. “As you have seen when Luca calls his cat he is always happy” (meaning 2). On the left, this is the matched version (i.e., the audio and the visual inputs match) whereas on the right this is the mismatched version (i.e., the audio of meaning 1 is aligned with the visual input of meaning 2). The left and right edges of gesture sequences and those of utterances were aligned.

We video recorded two native speakers of Italian—a male and a female—uttering ten different ambiguous Italian sentences (see Table 1). The speakers were unaware of the purpose or the specifics of the experiments. The speakers were asked to convey to an Italian listener the different meanings of the sentences using spontaneous gestures in the most natural way possible. They were video recorded under experimental conditions (i.e., not in natural setting) uttering the different sentences presented in Table 1 with each of their two different meanings. The co-speech gestures produced contained both iconic gestures (i.e., gestures expressing some aspects of the lexical content) and beats ones (i.e., gestures linked to some prosodic aspects of the utterance) gestures (see Kendon, 1994 for a review; McNeill, 1992). The videos of the speakers were framed so that only the top of their body, from their shoulders to their waist, was visible (see Movies S1, S2). Thus, the mouth—i.e., the verbal articulation of the sentences—was not visible. Two categories of videos were created from these recordings using Sony Vegas 9.0 software. One category corresponded to the “matched videos” in which the speakers' gestures and their speech matched and the second category corresponded to the “mismatched videos” in which the gestures were associated with the speech sound of the same sequence of words, but with the alternative meaning. To do so, we edited the original recordings and switched the acoustic and visual stimuli. This manipulation was not perceived by the participants as reported in the debriefing session. Then the gestures signaled the opposite meaning of that is signaled by the sentence for this condition. A total of 80 videos were created (each of the sentences was uttered twice). We ensured that, in the mismatched audio-visual presentations, the left and the right edges of the gesture sequences were aligned with the left and the right edges of the utterances (see Figure 1). This is an important point as in sign languages manual alignment with the signing stream is quite strict (Nespor and Sandler, 1999; Sandler, 2012) and co-speech gestures in general are tightly temporally linked to speech (McNeill et al., 2000). To remove the intelligibility of speech but to preserve prosodic information, the speech sounds were low-pass filtered using Praat software with the Haan band filter (0–400 Hz). As a result it was not possible to detect from speech which of the two meanings of a sentence was intended, as reported by the participants at the end of the experiment. The resulting stimuli had the same loudness of 70 dB.

### Procedure

Participants were tested in a soundproof room and the stimuli were presented through headphones. They were instructed to watch the videos and answer—by pressing a key on a keyboard—whether what they saw matched or mismatched what they heard (i.e., [S] = yes or [N] = no). A final debriefing (i.e., we explained the goals of the study) ensured that none of the participants understood the meaning of the sentences.

### Results and discussion

**Figure 2:**
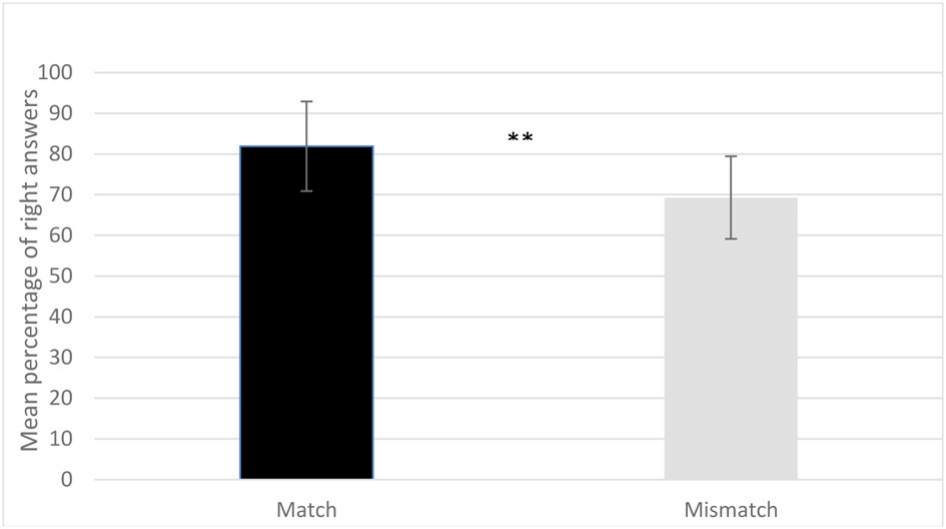
**Mean percentage of right answers in the match and mismatch conditions of Experiment 1**. Participants' mean percentage of right answers is significantly higher in the matching condition than in the mismatching one (^**^p < 0.0001). Errors bars represent the standard deviation.

The results show that participants correctly identified the videos in which hand gestures and speech matched [*M* = 81.9, *SD* = 11.03: *t*-test against chance with equal variance not assumed *t*_(19)_ = 12.93, *p* < 0.0001] and those in which they did not match [*M* = 69.3, *SD* = 10.17; *t*_(19)_ = 8.41, *p* < 0.0001]. A repeated measure ANOVA with condition (Match, Mismatch) and type of prosodic contour (IP and PP) was performed on the mean percentage. The ANOVA only revealed a significant main effect for condition [*F*_(1, 19)_ = 12.81, *p* = 0.002, ή^2^ = 0.4], but neither for type of prosodic contour [*F*_(1, 19)_ = 1.20, *p* = 0.287, ή^2^ = 0.06] nor for an interaction of type and condition [*F*_(1, 19)_ = 3.52, *p* = 0.076, ή^2^ = 0.16]. Participants answered correctly more often in the matching condition, and there are more errors for the mismatching one. In other words, they are more likely to incorrectly accept a mismatching video than to reject a matching one. A possible interpretation for this asymmetric results is that participants may detect some incoherences in the mismatching videos and these could lead them to a certain degree of uncertainty in their answers. To sum up, the results show that adult listeners detect the congruency between hand gestures and the acoustic speech signal even when only the prosodic cues are preserved in the acoustic signal (see Figure 2). The spontaneous gestures that accompany speech must therefore be aligned with the speech signal, suggesting a tight link between the motor-programs responsible for producing both speech and the spontaneous gestures that accompany it. The results of Experiment 1 thus show that adult listeners are sensitive to the temporal alignment of speech and the gestures that speakers spontaneously produce when they speak. In the next Experiment we asked whether the gestures that accompany speech have any effect on adult listeners' understanding of ambiguous sentences.

## Experiment 2

In sign languages, a good deal of prosodic information is conveyed by gestures of different parts of the face and body (Sandler, 2012). This information alone can distinguish coordinate from subordinate sentences and declarative sentences from questions (Pfau and Quer, 2010; Dachkovsky et al., 2013). This may suggest that in spoken languages too, listeners can actively use gestures accompanying speech for perceiving, processing and also understanding speech. For example, if gestures are carrying prosodic information about the grammatical structure of the speech signal, it should be easier for listeners to disambiguate a sentence that can have two different meanings when the gestures accompanying speech are visible and match the audible utterance. Experiment 2 was designed to test this hypothesis. We presented to Italian-speaking adults potentially ambiguous Italian sentences in which the audio-visual information was either matched or mismatched.

### Methods

#### Participants

We recruited 20 native speakers of Italian (9 females and 11 males, mean age 23 ± 3) from the subject pool of SISSA—International School of Advanced Studies (Trieste, Italy). Participants reported no auditory, vision, or language related problems. They received monetary compensation.

#### Stimuli

The same videos of the speakers recorded for Experiment 1 were used. However, for Experiment 2, the speech sound was not low-pass filtered (see Movies S3, S4). We added also audio-only samples of the sentences as a control condition. Thus, there were three categories of stimuli for Experiment 2: auditory only, auditory with matched gestures and auditory with mismatched gestures. For each of the categories, there were 10 different sentences (i.e., the same sentences as in Experiment 1) that could have two different meanings, uttered by a male and a female speaker. Thus, a total of 120 stimuli were created. We ensured that the left and right edges of gesture sequences and those of utterances were aligned. Speech sounds for all the stimuli had the same loudness of 70 dB.

### Procedure

Participants were tested in a soundproof room with headphones. They were instructed to both listen to and to watch the stimuli. After each presentation, a question appeared on the screen regarding the meaning of the sentence they had just perceived. For example, after “Quando Giacomo chiama suo fratello è sempre felice” (When—Giacomo—calls—his—brother—is—always - happy) either the question “Giacomo è felice?” *(Is Giacomo happy?)*, or the question “Suo fratello è felice?” *(Is his brother happy?)* appeared. Participants had to answer, by clicking on a keyboard, if the answer to the question was *yes* or *no*. In each of the three within-subject conditions (audio only, audio and gestures match, audio and gestures mismatch) participants saw 5 of the 10 sentences (total 10 different meanings) so that each meaning was paired with a “yes” question (“yes” = hit/“no” = miss) and a “no” question (“yes” = correct rejection/“no” = false alarm). Each participant heard the same sentence produced by the female and the male speaker resulting in a total of 120 trials.

### Results

**Figure 3:**
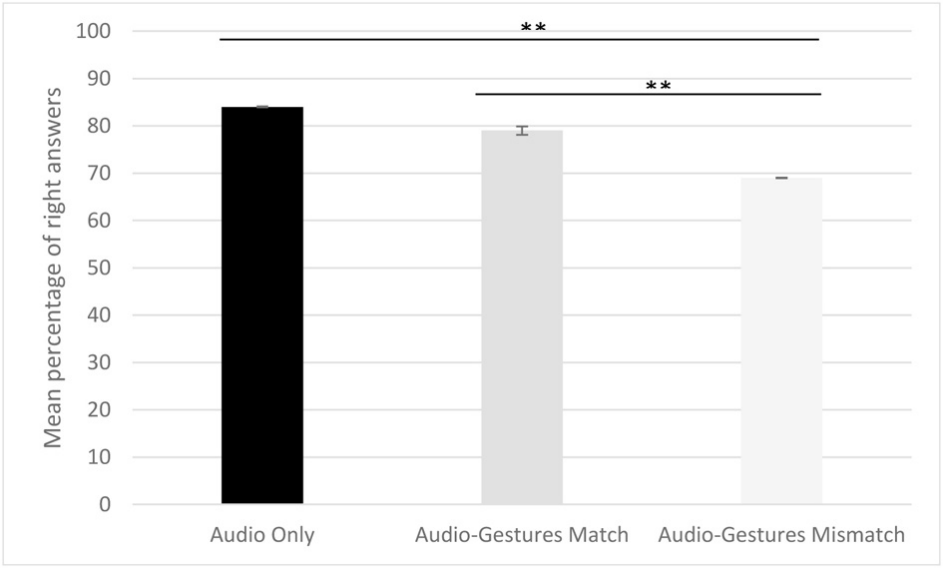
**Mean percentage of right answers in the audio only, the match and mismatch conditions of Experiment 2**. Participants' mean percentage of right answers is higher in the audio and matching conditions than in the mismatching one (^**^p < 0.0001). Errors bars represent the standard deviation.

First, comparisons against chance indicated that participants' overall accuracy of the presented stimuli was significantly above chance (see Figure 3) [Audio condition: *M* = 84.1, *SD* = 9.2: *t*-test against chance with equal variance not assumed *t*_(19)_ = 24.7, *p* < 0.0001; Match condition: *M* = 79, *SD* = 8.8, *t*_(19)_ = 23.5, *p* < 0.0001; Mismatch condition: *M* = 69.1, *SD* = 5.2, *t*_(19)_ = 31, *p* < 0.0001]. In order to determine participants' performance in each of the three conditions we calculated the F-score (2^*^accuracy^*^completeness)/(accuracy+completeness): the harmonic mean of Accuracy [#hits/(#hits+#false alarms)] and Completeness (#hits/(#hits+#misses)). We ran a repeated measures ANOVA with Condition (Audio Only, Audio-Gesture Match, Audio-Gesture Mismatch) and Type of Prosodic Contour (IP and PP) as within-subject factors. We found a significant main effect for condition [*F*_(2, 18)_ = 20.1, *p* = 0.0001, ή^2^ = 0.7], a marginally significant effect for Type [*F*_(1, 19)_ = 4.226, *p* = 0.054, ή^2^ = 0.18] and a significant interaction of Type and Condition [*F*_(2, 18)_ = 14.624, *p* < 0.0001, ή^2^ = 0.6]. Paired sample *t*-tests used for *post-hoc* comparisons (Bonferroni correction *p* < 0.0083) revealed a significant difference between Audio Only (*M* = 84.1, *SD* = 9.2) and Audio-Gestures Mismatch (*M* = 69.1, *SD* = 5.2) conditions [*t*_(19)_ = 6.78, *p* < 0.0001], and between Audio-Gesture Match (*M* = 79, *SD* = 8.8) and Audio-Gesture Mismatch conditions [*t*_(19)_ = 4.67, *p* < 0.0001], but not between Audio only and Audio-Gesture Match conditions [*t*_(19)_ = 1.40, *p* = 0.178]. While the type of the prosodic contour did not affect participants' performance in the Audio only condition [*M*_IP_ = 87, *SD*_IP_ = 10; *M*_PP_ = 79, *SD*_PP_ = 13: *t*_(19)_ = 2.408, *p* = 0.026], participants performed significantly better on sentences disambiguated with PP than on sentences disambiguated with IP boundaries in Audio-Gesture Match [*M*_IP_ = 75, *SD*_IP_ = 11; *M*_PP_ = 85, *SD*_PP_ = 12: *t*_(19)_ = −3.105, *p* = 0.006] and Audio-Gesture mismatch [*M*_IP_ = 64, *SD*_IP_ = 8; *M*_PP_ = 70, *SD*_PP_ = 10: *t*_(19)_ = −3.376, *p* = 0.003] conditions. First, these results show that matching gestures do not lead to a better comprehension than audio alone, while mismatching gestures hinder comprehension. Second, when the prosody of gestures mismatched that of speech, participants could not ignore the mismatch in their effort to disambiguate sentences. Interestingly, while on the whole, perceiving speech with and without gestures did not appear to influence sentence comprehension as scores are above chance level, participants have more difficulties to disambiguate sentences with IP than with PP boundaries both in the gestures matched and in the gestures mismatched conditions.

## General discussion

Our findings show that when presented with acoustic linguistic stimuli that contain only prosodic information (i.e., low-pass filtered speech), participants are highly proficient in detecting whether speech sounds and gestures match. The prosodic information of spoken language must therefore be tightly connected to gestures in speech production that are exploited in speech perception. The syntactic structure and the meaning of utterances appear thus not to be necessary for the perceiver to align gestures and prosody. Additionally, participants could also use co-speech gestures in their comprehension of potentially ambiguous sentences, i.e., sentences with the same sequence of words, thus totally ambiguous in their written form, but with different prosodic structures. The disambiguation of these sentences could be triggered either by the PP or by the IP division into constituents. Our results show that matching gestures do not lead to a better comprehension than audio alone, while mismatching gestures led participants to choose significantly more the meaning signaled by gestures. Therefore, gestures are used in interpreting the meaning of ambiguous sentences. Interestingly, in the presence of gestures, participants have more difficulties to disambiguate sentences with IP than with PP boundaries in both conditions. These results suggest that the presence of gestures impairs performances when auditory cues are stronger. For example, it is possible that PPs are less marked by auditory cues than the IPs and therefore gestures might give additional information in this case. It seems also important here to point out the fact that in the present study what we call mismatch videos are videos in which the audio file of one meaning of a sentence is presented with the image video of the alternative meaning of the same sentence. Therefore, this manipulation of stimuli could have led to a possible artifact in the participants' performances. Though this possibility cannot be excluded entirely, we believe it is unlikely. At the end of the test session, we asked participants whether they had noticed the mismatching manipulation. None of the participants tested reported any perception of a manipulation. Thus, when they had the two categories of sentences, matched and mismatched, they did not detect that they were different because one was manipulated and not the other.

As opposed to the visual perception of speech in the speakers' face, where the movements of the mouth, the lips, but also the eyebrows (Krahmer and Swerts, 2004) are unavoidable in the production of spoken language, the gestures that accompany speech belong to a different category that is avoidable in speech production. Even though mismatching gestures decrease the intelligibility of spoken language, the addition of matching gestures does not appear to give an advantage over speech perception in the auditory modality alone. We are, in fact, able to understand the meaning of sentences when talking on the phone, or if our interlocutor is for other reasons invisible. Our results, however, suggest that the prosody of language extends from the auditory to the visual modality in speech perception.

This link between speech and gestures is congruent with neuropsychological evidence for a strong correlation between the severity of aphasia and the severity of impairment in gesturing (Cocks et al., 2013). While further studies are clearly needed to identify the specific aspects of spontaneous gestures that are coordinated with speech acts, our results demonstrate that part of speech perception includes the anticipation that bodily behaviors, such as gestures, be coordinated with speech acts. Prosodic Phonology thus appears—at least in part—not to be a property exclusive to oral language. In fact, it has abundantly been shown to characterize also sign languages where it has an influence on all body movements (Nespor and Sandler, 1999; Wilbur, 1999; Sandler, 2011, 2012). It is also—at least in part—not specific to language. Previous findings have shown that part of prosody, i.e., rhythmic alternation as defined by the Iambic—Trochaic Law (Bolton, 1894; Nespor et al., 2008; Bion et al., 2011) characterizes also the grouping of non-linguistic visual sequences (Peña et al., 2011). Thus, language is a multimodal experience and some of its characteristics are domain-general rather than domain-specific.

### Conflict of interest statement

The authors declare that the research was conducted in the absence of any commercial or financial relationships that could be construed as a potential conflict of interest.

## Footnotes

^1^Though recursive prosodic phrasal constituents have been proposed at the level of the Intonational Phrase (Ladd, 1986) we rely on the more standardly accepted view that phrasal prosody has no recursive constituents (Selkirk, 1984; Nespor and Vogel, 1986, 2007).

## Supplementary material

Movie S1 **One sentence low-pass filtered speech, match condition (Experiment 1)**.

Movie S2 **Same sentence low-pass filtered speech, mismatch condition (Experiment 1)**.

Movie S3 **One sentence, match condition (Experiment 2)**.

Movie S4 **Same video, mismatch condition (Experiment 2)**.
